# Transcriptome in Combination Proteome Unveils the Phenylpropane Pathway Involved in Garlic (*Allium sativum*) Greening

**DOI:** 10.3389/fnut.2021.764133

**Published:** 2021-11-01

**Authors:** Jinxiang Wu, Zhonglu Niu, Xiaoming Lu, Xiaozhen Tang, Xuguang Qiao, Longchuan Ma, Chao Liu, Ningyang Li

**Affiliations:** ^1^Key Laboratory of Food Processing Technology and Quality Control in Shandong Province, College of Food Science and Engineering, Shandong Agricultural University, Tai'an, China; ^2^Garlic Science and Technology Research Center of Jinxiang, Jining, China; ^3^Key Laboratory of Novel Food Resources Processing, Ministry of Agriculture and Rural Affairs/Key Laboratory of Agro-Products Processing Technology of Shandong Province/Institute of Agro-Food Science and Technology, Shandong Academy of Agricultural Sciences, Jinan, China

**Keywords:** garlic, green discoloration, transcriptome, genome expression, phenylpropane pathway

## Abstract

Garlic (*Allium sativum*) is an important vegetable crop that is widely used in cooking and medicine. The greening phenomenon of garlic severely decreases the quality of garlic and hinders garlic processing. To study the mechanism of garlic greening, comprehensive full-length transcript sets were constructed. We detected the differences in greening between Pizhou (PZ) garlic and Laiwu (LW) garlic that were both stored at −2.5°C and protected from light at the same time. The results showed that 60,087 unigenes were respectively annotated to the NR, KEGG, GO, Pfam, eggNOG and Swiss Prot databases, and a total of 30,082 unigenes were annotated. The analysis of differential genes and differential proteins showed that PZ garlic and LW garlic had 923 differentially expressed genes (DEGs), of which 529 genes were up regulated and 394 genes were downregulated. Through KEGG and GO enrichment analysis, it was found that the most significant way of enriching DEGs was the phenylpropane metabolic pathway. Proteomics analysis found that there were 188 differentially expressed proteins (DAPs), 162 up-regulated proteins, and 26 down-regulated proteins between PZ garlic and LW garlic. The content of 10 proteins related to phenylpropanoid biosynthesis in PZ garlic was significantly higher than that of LW garlic. This study explored the mechanisms of garlic greening at a molecular level and further discovered that the formation of garlic green pigment was affected significantly by the phenylpropanoid metabolic pathway. This work provided a theoretical basis for the maintenance of garlic quality during garlic processing and the future development of the garlic processing industries.

## Introduction

Garlic is a popular condiment in the world. It is not only rich in nutrients, but also has many pharmacological effects, such as anti-bacterial, anti-inflammatory, anti-oxidation, anti-tumor, hypoglycemic, lipid-lowering and immune regulation (1–3). When it is processed into garlic products, such as granules, powder, minced paste, oleoresin, and puree, garlic is easy to turn green, which despite that it being necessary for the traditional Chinese food “Laba garlic”, it seriously affects the appearance and quality of products, limits the commercial use of products, and reduces the economic value of garlic (4, 5).

The mechanism of garlic green discoloration is always a research hotspot that can be divided into four steps. The first step is a very fast enzymatic reaction in which S-(1-propenyl)-L-cysteine sulfoxide (1-PeCSO), the precursor of flavor, produces water-soluble and volatile 1-propenyl containing thiosulfinate or di(1-propenyl) thiosulfinate under the catalysis of alliinase (6). In the second step, di(1-propenyl) thiosulfinate reacts very slowly with amino compounds (amino acids, peptides and proteins) to form pigment precursor (PP)-pyrrolyl amino acids (7). The third step is the conversion of S-(2-propenyl)-L-cysteine sulfoxide (2-PeCSO) into di(2-propenyl) thiosulfinate by alliinase. Finally, blue pigment is formed by the reaction of thiosulfinate and PP. The unstable blue pigment will be slowly decomposed into yellow pigment. The reaction of pyruvic acid with PP [2-(1H-pyrrolyl) carboxylic acids] might serve as another pathway for forming the yellow pigment (8), which is mixed with the undecomposed blue pigment to form the green pigment as the fourth step (5).

The side chain of 2-(1H-pyrroliyl) carboxylic acid plays a critical role in garlic greening. The hydrophilic and hydrophobic chains of 2-(1H-pyrrole) carboxylic acid have different greening effect on garlic (9, 10). In addition, low temperature and pH have important effects on garlic green pigment. Low temperature storage is conducive to breaking dormancy of garlic bulbs, leading to the accumulation of garlic precursor substances (1-PECSO), which is an important compound participated in discoloration (6, 7). The optimal pH value for garlic greening is about 5.5. But the formation of blue pigment leads to the change of pH value, and the absorbance at 590 nm is inversely proportional to the change of pH value (11). Zhao et al. (12) found that the decomposition of blue pigments was inhibited by the acidic environment (pH 5.0). The alkaline conditions could accelerate the decomposition of blue pigments in garlic compared with garlic homogenate at pH 6.5.

High-throughput sequencing (RNA-seq) is widely used in the study of transcriptional levels. It can detect the transcriptional levels of genes in different tissues under different conditions, and observe the changes and responses of genes to different abiotic stresses. Transcriptome analysis is also applied to research marker development, gene transcription, mining of functional genes, gene structure, and evolution. At present, the transcriptome of garlic has been sequenced and reassembled (13). Kong et al. (14) studied the long-term adaptation response to salt stress during garlic growth and found that brassinosteroid might regulate lignin biosynthesis at the transcriptional level. Rukmankesh et al. (15) studied the transcriptomics of snow mountain garlic, screened out candidate genes related to organic sulfur metabolism in garlic bulbs, and analyzed the highly expressed genes involved in photosynthesis in garlic leaves. Li et al. (16) studied the effect of low temperature treatment on the green discoloration of garlic at the transcription level. Through the analysis of DEGs, they proved that the alliinase coding gene, γ-glutamyl transpeptidase (GGT) coding gene, and δ-aminolevulinic acid dehydratase (ALAD) coding gene might be involved in garlic greening.

At present, there are few reports on the cause of garlic greening at the level of gene and protein. PZ and LW garlic are both large with white skin and crisp meat but their spicy intensity, dormancy, and storage resistance are all different (17, 18). This study explored the potential mechanism of green discoloration of different garlic varieties. The genome-wide and transcriptome analysis was performed, and the differential transcription and expression of genes and proteins in PZ garlic and LW garlic were obtained. Through enrichment analysis, the garlic green change was located in the phenylpropane metabolic pathway. This study provided a new perspective on the mechanism of green discoloration of garlic.

## Materials and Methods

### Materials

The raw garlic was top-grade white produced from Laiwu, Shandong Province, and Pizhou, Jiangsu Province of China. The PZ garlic was *pizhoubaisuan* and the LW garlic was *Baipizajiaosuan*. The garlic was big with white skin, crispy flesh, moderate spicy taste, and neat shape, which made it resistant to transportation and stored at −2.5°C in the dark for the same time. Garlic had no mechanical damage and biological disease.

Cell lysis buffer (P0013), sodium pyrophosphate, EDTA, β-glycerophosphate, phosphate-buffered saline (PBS), Folin-Ciocalteu, methanol, acetic acid, and H_3_PO_4_-KH_2_PO_4_ buffer (HPLC) were ordered from Beijing Solarbio Technology Co., Ltd (China). Formic acid and acetonitrile were of chromatography grade. All other reagents used were of analytical grade.

### Extraction of Garlic Green Pigment

Mixed PZ and LW garlic cloves (20 g) were utilized to determine the extent of discoloration, and the potential spectrometric interference was minimized by removing the green sprouts. Then, 5 ml of 2% citric acid solution was thoroughly mixed with the minced sprout-free garlic samples to incubate for 30 min at 80°C. Afterward, different volumes of 95% ethanol was added to the resultant mixture after lowering to room temperature, succeeded by 24 h-incubation at 4°C (19). The supernatant was scanned for the full spectrum.

### Plant Treatments and RNA Isolation

Pizhou garlic and LW garlic were stored at −2.5°C in dark condition for 30 days. Three biological replicate samples were taken from each of the two groups of garlic cloves, followed by immediate freezing in liquid nitrogen and stored at −80°C. The frozen garlic bulb samples stored in a refrigerator at −80°C were taken out and crushed, placed in a precooled mortar and ground with liquid nitrogen. Gene JET Plant RNA Purification Mini Kit (#K0801) was used to extract total RNA from 50 mg tissue of each sample according to the instructions of the manufacturer. Then, the extracted RNA was detected by Qubit 2.0 RNA Wide Domain Detection Kit (Invitrogen, USA) and NanoDrop 2000 (Thermo Fisher Scientific, USA) for precise quantitative detection (20). The integrity of RNA was evaluated using an Agilent Bioanalyzer 2100 (Agilent Technologies, USA).

### Construction and Sequencing of PacBio Library

The Iso-Seq library was constructed by mixing the qualified RNA from all samples in equal quantity. First, SMARTer^®^ PCR cDNA Synthesis Kit was used to reverse-transcript the mixed RNA sample. The KAPA HiFi PCR Kit was used to perform PCR amplification. The PCR products with the length of.5 to 6.0 kb were selected using BluePippin Size Selection system (Sage science, USA). Subsequently, the PCR products were used as template to construct the SMRTbell library with the SMRTbell template pre Kit 1.0. For each SMRT cell, PacBio Sequel platform with P6-C4 polymerase was used to sequence a total of 30 ng of the library.

### Analysis of PacBio Iso-Seq Reads

We used the IsoSeq v3 software to analyze raw polymerase reads. First, the post-filter polymerase reads were obtained by taking out the low-quality data and adapters. Then, the circular consensus sequence (reads of insert, ROI) was created from the subread BAM files (21). According to 3′ or 5′primer, we further classified the transcript sequences of full-length (FL) and non-full-length (nFL) from all the ROIs, and poly A tails were simultaneously observed. The software of CD-Hit was used to remove the redundancy of FLs with parameter-c.9 (22). Finally, the highly accurate nonchimeric full-length genes (isoform level) were created for the subsequent analysis after correction.

### Transcriptomic Analysis and Function Annotation

All unigenes were annotated from public databases, including the Kyoto Encyclopedia of Genes and Genomes (KEGG) database, NCBI non-redundant protein sequences (NR) database, Gene Ontology (GO) database, Non-supervised Orthologous Groups (eggNOG) database, protein family (Pfam) database, and Swiss-Prot protein database.

### Illumina Library Construction and RNA Sequencing

The Illumina libraries were constructed using Illumina TruSeq RNA Library Preparation Kit v2 based on the instructions of the manufacturer. Agilent 2,100 Bioanalyzer was used to analyze the quality and quantity of cDNA libraries. The Illumina sequencing platform (HiSeqTM 4000) was used to perform the sample library sequencing (23).

The FastQC tool (http://www.bioinformatics.babraham.ac.uk/projects/fastqc/) was used to check the quality of the sequencing data. The clean reads were generated by performing Trimmomatic software to remove the adaptor and low-quality sequences (24). Bowtie2 v2.4.4 software was used to map high quality reads to reference sequences generated by PacBio (25).

### Analysis of Differentially Expressed Genes

The expression value of the Illumina reads of each sample was calculated using the full-length isoform transcripts obtained by performing SMRT Iso-Seq analysis as reference sequences via RSEM with default parameters (26). Gene expression levels were quantified using the fragments per kilobase of exon per million mapped fragments (FPKM) (27). The R package DESeq2 was used to identify the DEGs with the criteria of fold change > 2 and q < 0.05 (28). The top GO package and KOBAS (version 3.0) were used to perform GO and KEGG enrichment analyses, respectively. And GO categories or pathways with *p* < 0.05 were thought to be significantly enriched (29).

### Protein Extraction and Quantification

Pizhou and LW garlic samples (60 mixed individuals in each sample) were used to extract protein with cell lysis buffer (P0013) containing 20 mM Tris (pH 7.5), 1% Triton X-100, 150 mM NaCl, and various inhibitors including β-glycerophosphate, EDTA, and sodium pyrophosphate. The resulting protein was quantified with label-free quantitation and MaxQuant software was used to analyze quantitative information of peptides generated by LC-MS detection to obtain the relative content of a corresponding protein. Perseus software was used to generate the differentially abundant proteins (DAPs) (30).

### Quantitative Reverse-Transcription Polymerase Chain Reaction (qRT-PCR) Validation

To verify the reproducibility and reliability of the RNA-seq data, we randomly selected 10 up-regulated DEGs in the phenylpropane biosynthesis pathway (c11315/f1p23/2741, c116256/f2p5/1450, c174936/f2p3/1715, c261046/f2p6/2013, c3369/f1p14/2256, c44043/f1p3/1144, c61149/f1p35/2029, c70286/f1p12/1308, c86756/f1p3/1001, c88562/f1p10/1325) from the RNA-seq sequencing results for qRT-PCR verification. A gDNA eraser (Clontech Laboratories, USA) was used to remove the DNA in the RNA while the purified RNA was reverse transcribed using Takara PrimeScript RT Kit, and the cDNA obtained was used for real-time fluorescence quantification (qRT-PCR). Gene-specific primers were designed using Primer Premier 5.0 software (Supplementary Table 1). The qRT-PCR experiment was performed using Maxima SYBR Green qPCR Master Mix (Thermo Fisher Scientific) Kit and was completed on the Applied Biosystems 7500 fast real-time PCR system using *actin* as a reference gene (14). The 2^−ΔΔCt^ method was used to calculate the expression of differential genes, and each treatment was repeated 3 times (31).

### Analysis of Metabolites in Phenylpropane Pathway

#### Analysis of Total Polyphenols

The total polyphenols contents of PZ garlic and LW garlic were measured by the following methods.

Two grams of crushed garlic were added with water for a final volume of 50 ml, mixed well and left to stand for half an hour. Then, 1ml of supernatant was mixed evenly with 0.5 ml Folin-Ciocalteu reagent. After 5 min, 1.5 ml of 10% Na_2_CO_3_ solution was added and fixed the volume to 10 ml with deionized water. This mixed solution was then placed in a 75°C water bath or 10 min and, afterward, allowed to stand at room temperature for 60 min. Afterwards, its absorbance at 765 nm was measured against a blank reagent using gallic acid as the standard. Each procedure was repeated three times (32).

#### Analysis of Phenolic Acids

The following methods were used to measure the total (free and bound) phenolic acids of PZ garlic and LW garlic.

Seventy-five grams of peeled garlic cloves was homogenized in a blender with a mixture of 75 ml methanol (containing 2 g/L of butylated hydroxyanisole) and 10% acetic acid (85:15). The garlic homogenate was ultrasonicated for 30 min. Then, the samples were alkaline hydrolyzed by adding 50 mL of deionized water containing 22 mM ethylenediaminetetraacetic acid (EDTA) and 7.5 mL of deionized water containing 2% ascorbic acid and 25 ml of 10 M NaOH solution. This mixture was incubated at 30°C for 30 min. The solution was then adjusted to a pH of 2 with 6 M HCl, and the phenolic acids were extracted three times with 150 mL of ethyl acetate. The combined extracts were blown dry and dissolved in 10 ml of methanol. Finally, the samples were filtered and injected into the HPLC. Each procedure was repeated 3 times (33).

The high-performance liquid chromatography with diode array detector system apparatus (Shimazu, Tokyo, Japan) coupled with an Agilent C18 column (250 mm × 4.6 mm, 5 μm) was used to separate the phenolic Acids and measured the relative contents of phenolic Acids. The mobile phases consisted of solvent A (0.1% formic acid in water) and solvent B (100% acetonitrile) at a 1 ml/min flow rate. A gradient elution program was used as follows: 0–8 min, 0–12% B; 8–30 min, 12–50% B; 30–40 min, 50–100% B; 40–45 min, 100–100% B; 45–55 min, 100–12% B; and 55–60 min, 12–12% B. The column temperature was maintained at 25°C and the injection volume was 20 μl. The detection wavelengths were set to be 280 nm.

## Results

### Green Discoloration of PZ Garlic and LW Garlic

**Figure 1:**
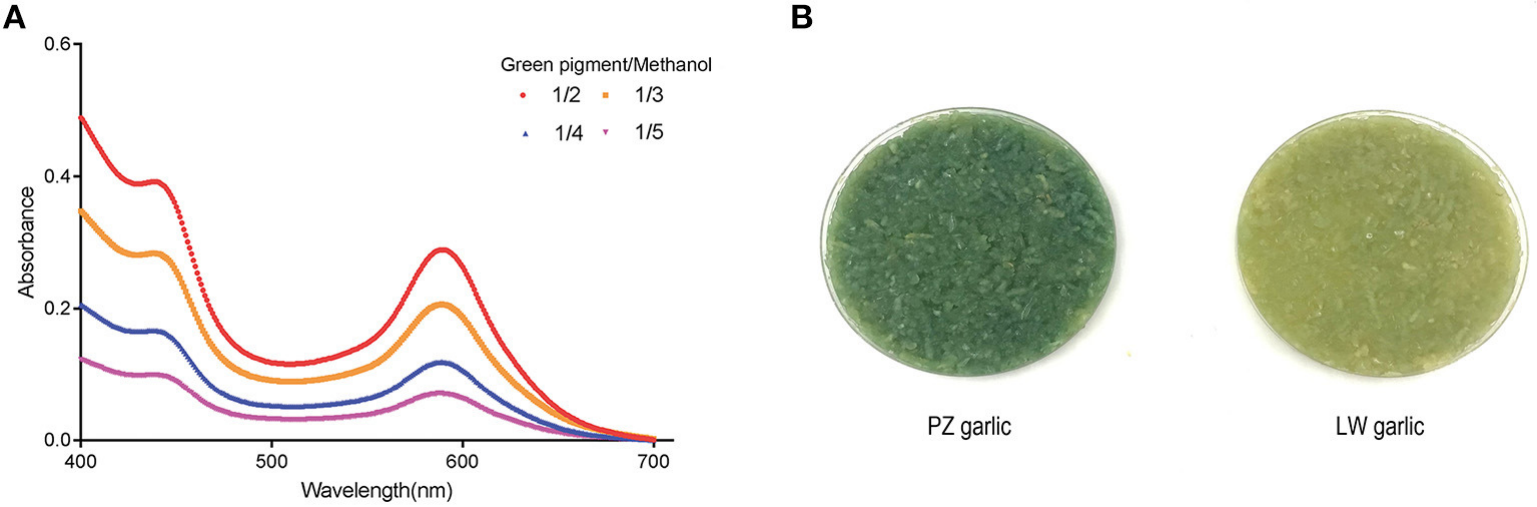
Extraction of garlic green pigment. **(A)** Measuring absorption spectrum of garlic green pigment. Mixed samples of Pizhou (PZ) garlic and Laiwu (LW) garlic were used for green pigment measurement. 440 nm indicated yellow pigment crests and 590 nm indicated blue pigment crests. **(B)** Green color change of PZ garlic and LW garlic. PZ garlic on the left and LW garlic on the right.

Two absorbance maxima at 440 and 590 nm were indicated in the absorption spectrum of a methanol extract with garlic green pigment in the previous studies (5, 10, 34). We also monitored the absorbance of crude methanol extracts containing pigment at different concentrations to support this observation. The absorbance maxima of green pigment remained at 440 and 590 nm, although the green pigment concentration varied (Figure 1A) (35). Because our conclusions were consistent with previous reports, we proceeded to determine the degree of garlic greening using the absorbance maxima 440 and 590 nm as indicators.

Pizhou garlic and LW garlic were stored at −2.5°C in dark condition for 30 days. Then, storage conditions were adjusted to 4°C for 15 days to break dormancy, and green discoloration difference between PZ garlic and LW garlic were observed. With the extension of cold treatment time, the absorbance values at 440 and 590 nm of two groups of garlic increased along with their degree of green change, but the degree of green change of PZ garlic was always higher than that of LW garlic (Figure 1B).

### Transcriptome Assembly and Function Annotation

**Figure 2:**
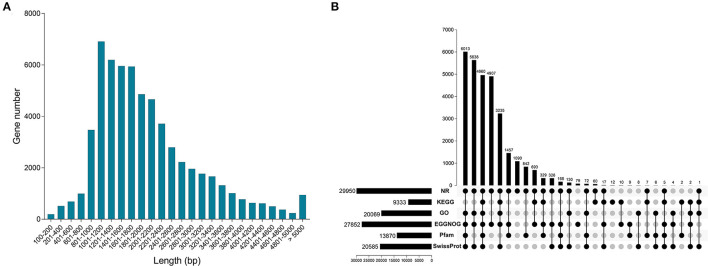
Characterization of full-length transcriptome. **(A)** The distribution of all gene length. **(B)** Functional annotation of all genes from NR, KEGG, GO, eggNOG, Pfam and SwissProt databases.

In this study, we obtained 60,876 unigenes. Distribution analysis of length showed that the number of unigenes between 1,001–1,200 bp was the largest, followed by 1,201–1,400 bp (Figure 2A). All unigenes were annotated from six databases, and there were 2,9950, 9,333, 20,069, 27,852, 13,870 and 20,585 unigenes significantly matched in the databases NR, KEGG, GO, eggNOG, Pfam and Swiss-Prot, respectively (Figure 2B). In the six databases, a total of 30,082 unigenes were functionally annotated to at least one database (Supplementary Table 2). In addition, a total of 4,960 unigenes were annotated to all databases (Figure 2B).

### DEG Identification, GO Enrichment Analysis, and KEGG Enrichment Analysis

**Figure 3:**
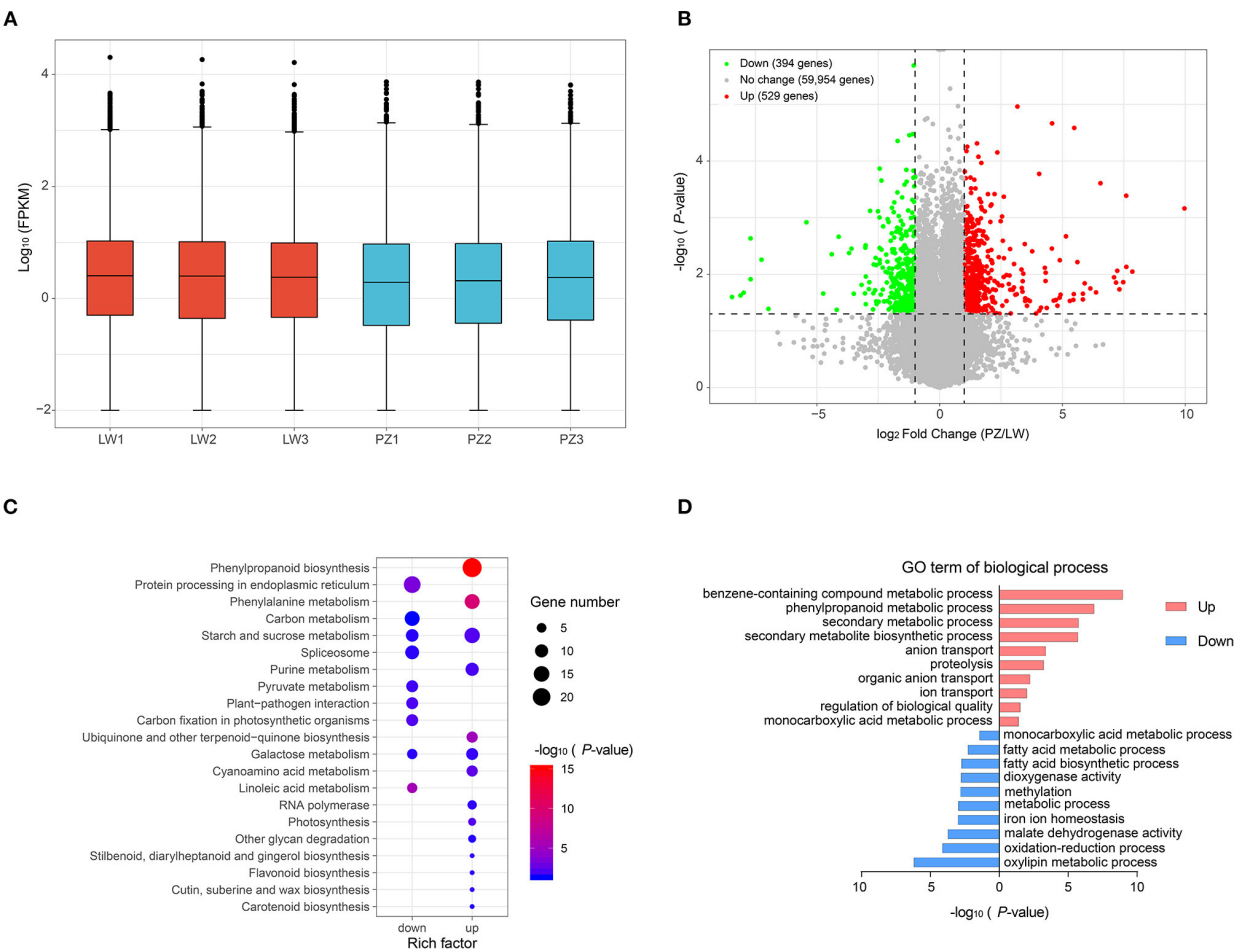
Identification and functional analysis of differentially expression genes (DEGs). **(A)** Expression level of all genes. **(B)** Volcano map showed the distribution of all differently expressed genes (DEGs). **(C)** KEGG pathways significantly enriched (*p*-value < 0.05) by up and down-regulated DEGs were shown. **(D)** The top 10 GO terms (rank by *P*-value) of biological process were showed.

We used FPKM to normalize gene expression (Figure 3A; Supplementary Table 3). As shown in Figure 3A, distribution of unigene expression showed that the expression patterns of all samples were similar. We performed differential expression analysis of PZ vs. LW based on FPKM value and unigene with *p* < 0.05. An absolute value of log2FC > 1 was defined as differential expression gene (DEG). A total of 923 DEGs, such as 529 up-regulated DEGs, and 394 down-regulated DEGs in PZ and LW garlic were observed (Figure 3B; Supplementary Table 4). KEGG enrichment analysis of DEGs was performed for further investigating functions, and 264 up-regulated DEGs were found to be enriched in 91 pathways and 138 down-regulated DEGs were enriched in 38 pathways in PZ garlic (Supplementary Table 5). There were 14 and 9 pathways, which were remarkably enriched (*p* < 0.05), in up and down DEGs, respectively (Figure 3C). Interestingly, up-regulated DEGs were remarkably enriched to phenylalanine metabolism, phenylpropanine biosynthesis, sucrose, and starch metabolism. In purine metabolism pathway, down-regulated DEGs were remarkably enriched in protein processing in carbon metabolism, endoplasmic reticulum, and spliceosome pathways (Figure 3C; Supplementary Table 5). GO term enrichment analysis of DEGs indicated that the up-regulated DEGs identified in this work were remarkably enriched into 143 GO terms, and downregulated DEGs were significantly enhanced into 74 GO items (Supplementary Table 6). The top 10 GO terms of Biological Process were shown in Figure 3D. The up-regulated DEGs were remarkably enriched in benzene-containing compound metabolic process, phenylpropanoid metabolic process, and secondary metabolic process pathways. The downregulated DEGs were remarkably enhanced in oxidation-reduction process, oxylipin metabolic process, and malate dehydrogenase activity pathways (Figure 3D).

### Characteristic Analysis

**Figure 4:**
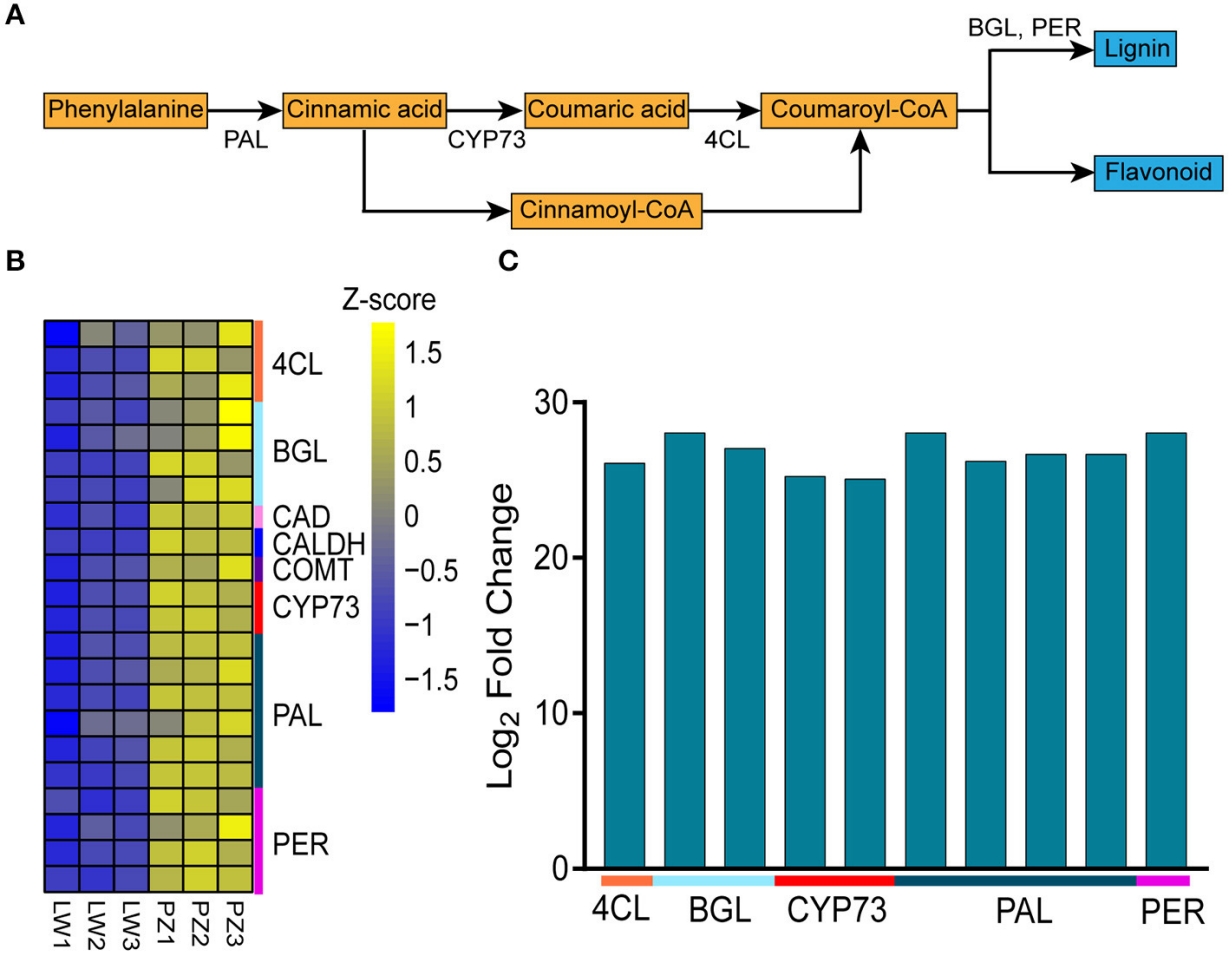
Differentially expression genes involved in Phenylpropanoid biosynthesis. **(A)** The key genes of Phenylpropanoid biosynthesis. **(B)** Expression patterns of differently expressed genes (DEGs) involved in Phenylpropanoid biosynthesis. The DEGs are 4-coumarate—CoA ligase (4CL), beta-glucosidase (BGL), cinnamyl-alcohol dehydrogenase (CAD), caffeic acid 3-O-methyltransferase (COMT), trans-cinnamate 4-monooxygenase (CYP73A), phenylalanine ammonia-lyase (PAL), peroxidase (PER), and coniferyl-aldehyde dehydrogenase (CALDH). **(C)** Differentially abundance proteins (DAPs) involved in Phenylpropanoid biosynthesis.

Since phenylpropane metabolism was significantly enhanced in up-regulated DEGs, we further explored the functions of DEGs in the pathway (Figure 4A). In Figure 4B, Z-score was used to show the expression patterns of genes and reflect the expression trend of genes in different samples (36, 37). It was found in KEGG enrichment analysis that 22 DEGs in phenylpropanoid biosynthesis pathway in PZ garlic were up-regulated compared with LW garlic (Figure 4B; Table 1). No downregulated differential genes were associated with this pathway, indicating that it might play an important role in the greening process of garlic.

**Table 1:** DEGs in the phenylpropanoid biosynthesis.

**Gene ID**	**Gene name**	**Desription**	**Regulation**
c61149/f1p35/2029	4CL	4-coumarate–CoA ligase	Up
c261046/f2p6/2013	4CL	4-coumarate–CoA ligase	Up
c9430/f1p36/1338	4CL	4-coumarate–CoA ligase	Up
c86756/f1p3/1001	BGL	Beta-glucosidase	Up
c24450/f1p15/3426	BGL	Beta-glucosidase	Up
c26268/f1p14/1850	BGL	Beta-glucosidase	Up
c32513/f1p279/2128	BGL	Beta-glucosidase	Up
c116256/f2p5/1450	CAD	Cinnamyl-alcohol dehydrogenase	Up
c11693/f1p13/1676	COMT	Caffeic acid 3-O-methyltransferase	Up
c88562/f1p10/1325	CYP73A	Trans-cinnamate 4-monooxygenase	Up
c70286/f1p12/1308	CYP73A	Trans-cinnamate 4-monooxygenase	Up
c3369/f1p14/2256	PAL	Phenylalanine ammonia-lyase	Up
c11315/f1p23/2741	PAL	Phenylalanine ammonia-lyase	Up
c1076/f3p52/2334	PAL	Phenylalanine ammonia-lyase	Up
c14606/f1p46/3127	PAL	Phenylalanine ammonia-lyase	Up
c87606/f1p10/1076	PAL	Phenylalanine ammonia-lyase	Up
c68310/f1p8/2095	PAL	Phenylalanine ammonia-lyase	Up
c44043/f1p3/1144	PER	Peroxidase	Up
c12491/f1p3/1162	PER	Peroxidase	Up
c22433/f1p2/1336	PER	Peroxidase	Up
c317700/f1p2/1273	PER	Peroxidase	Up
c174936/f2p3/1715	CALDH	Coniferyl-aldehyde dehydrogenase	Up

In order to study whether the results of transcriptome analysis affect the changes of protein level, we performed proteome analysis. Proteome analysis showed that a total of 188 differentially abundant proteins (DAPs) were identified in comparison of PZ vs LW, among which 162 DAPs were up-regulated and 26 DAPs were down-regulated. In the DAPs, there were 10 up-regulated DAPs related to phenylpropanoid biosynthesis (Figure 4C; Supplementary Table 7), which further verified the transcriptome results. Among 22 DEGs and 10 DAPs associated with phenylpropane pathway, 11 genes are common. Therefore, phenylpropanoid biosynthesis might play an important role during the process of garlic greening.

### Validation of the Differential Expression by qRT-PCR

**Figure 5:**
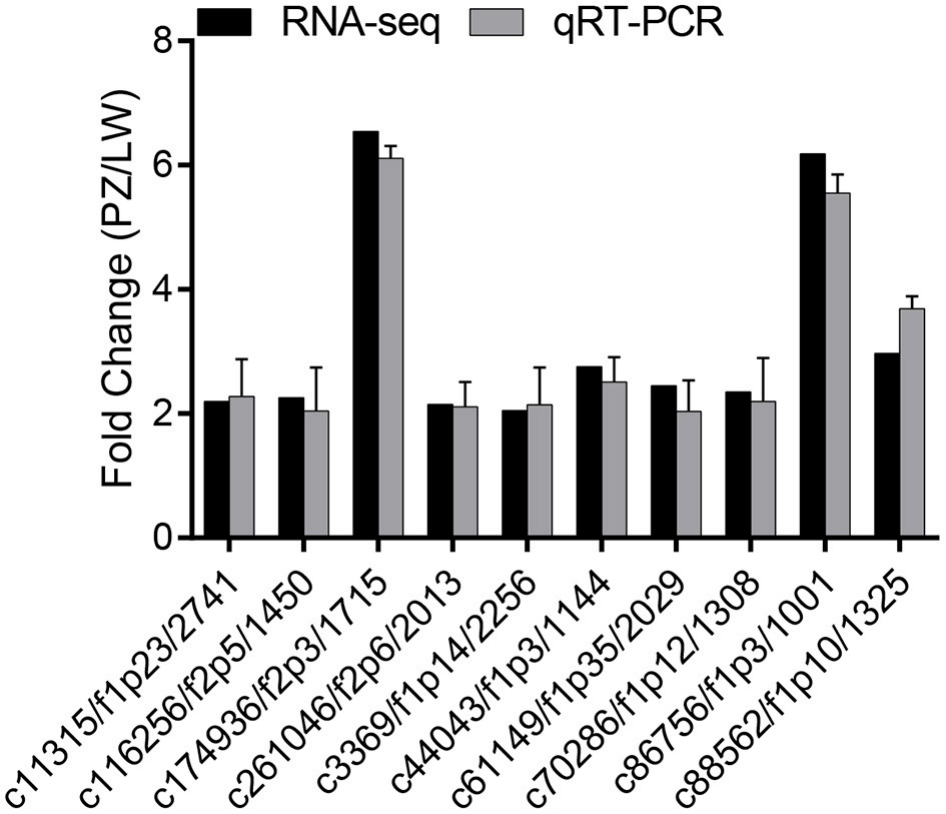
qRT-PCR was used to verify the RNA-seq data. The hist shown fold change of PZ/LW.

The expression levels of 10 up-regulated DEGs in phenylpropanoid biosynthesis pathway were verified by qRT-PCR for the change folds, which were consistent with the results of Illumina sequencing (Figure 5). The correlation between these two methods was.85. These results suggested that the expression proofing characterized by Illumina sequencing was reliable (38).

### Analysis of Compounds Involved in Phenylpropane Pathway

**Figure 6:**
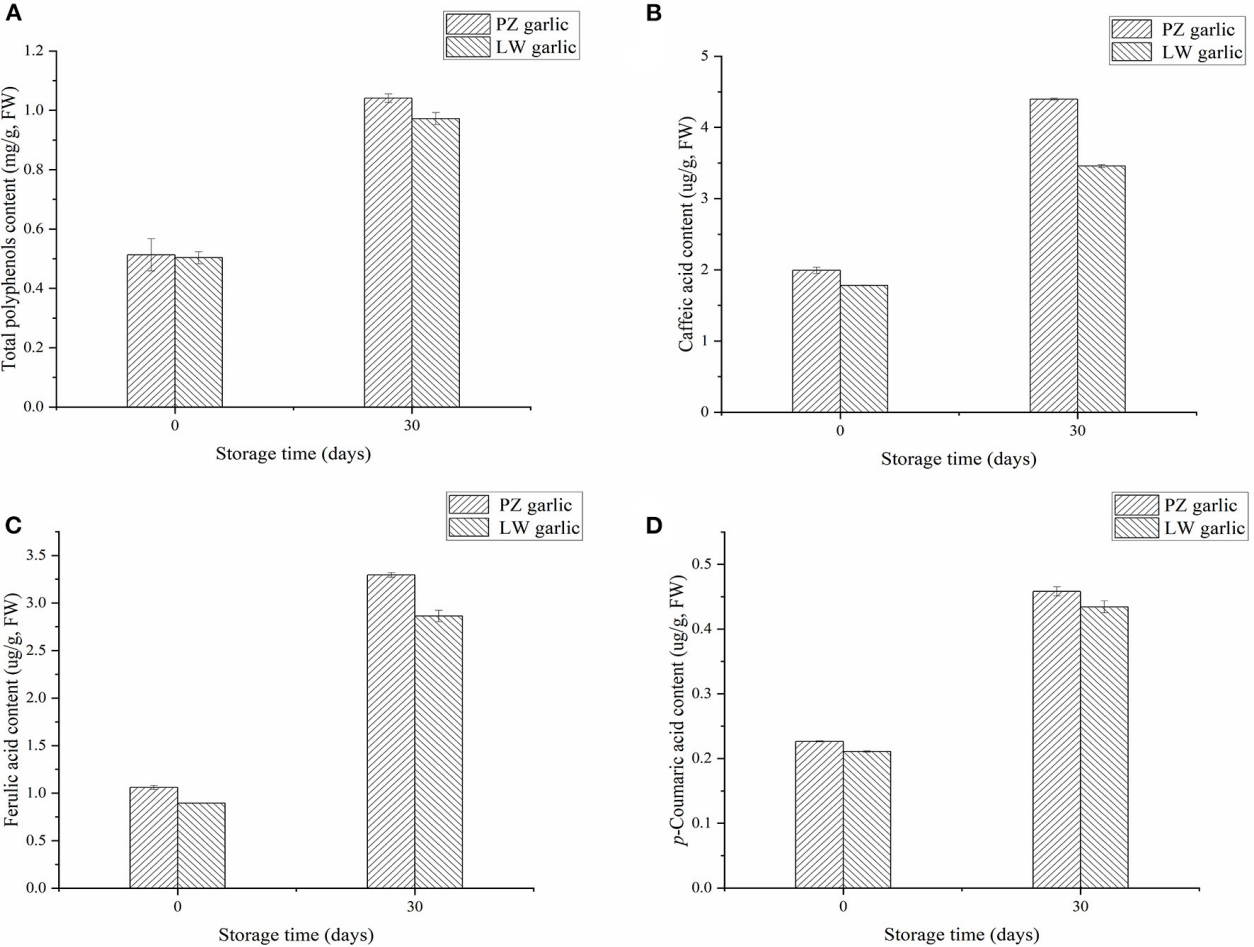
Relative contents of different compounds involved in phenylpropane pathway. **(A)** Total polyphenols content. **(B)** Caffeic acid content. **(C)** Ferulic acid content. **(D)** *p*-coumaric acid content.

We measured the relative contents of total polyphenols and phenolic acids in two groups of garlic, which were compounds of the phenylpropane pathway. We measured that the phenolic acids in garlic mainly include caffeic acid, ferulic acid, and *p*-coumaric acid. With the extension of garlic storage time at low temperature, the contents of total polyphenols, caffeic acid, ferulic acid, and *p*-coumaric acid increased (Figure 6). The contents of these compounds in PZ garlic were higher than that in LW garlic, which was consistent with the intensity of green discoloration, further confirming the RNA-seq data and conclusions.

## Discussion

Garlic discoloration is one of the important factors affecting the processing quality of garlic (39). In this study, it was found that PZ garlic and LW garlic turn green when stored at −2.5°C for the same time. This phenomenon was because GGT activity were increased during cold storage. GGT hydrolyzed peptides to produce isoallicin. The increase of isoallicin content led to the increase of green discoloration of garlic (40). In previous studies, ALAD and alliinase all played an important role in garlic greening besides GGT (41).

Through transcriptomic analysis of the green discoloration difference between PZ garlic and LW garlic, we found that the DEGs were significantly enhanced in the phenylpropane metabolic pathway. Phenylpropane metabolism was a key pathway in plant secondary metabolism and played an important role in the defense response to abiotic stresses such as high and low temperature, salt and drought, and ultraviolet and heavy metals (42). Secondary metabolites produced by this pathway include antimicrobial and antioxidant phenolic compounds and flavonoids, lignin, the structural barrier substance, phenolic acids, pigments, and other substances (43). Temperature has an important effect on phenylpropane pathway. Herrera et al. (32) studied the effects of cold treatment of garlic cloves before garlic planting and temperature as thermal time in accumulated growing degree days during plant growing on the growth and development of garlic. Low temperature treatment of garlic bulbs before planting can accelerate the harvest period of garlic and increase the content of total phenols and anthocyanins in the cataphylls of garlic bulbs. The interaction between low temperature treatment and planting date increased the concentration of total flavonoids. In this study, the enrichment of phenylpropane pathway showed the stress response of garlic to low temperature environment.

In this study, 22 up-regulated DEGs were significantly enriched in phenylpropane metabolic pathway, including three 4-coumarate-CoA ligase (4CL) encoding genes, four β-glucosidase encoding genes, and one cinnamyl-alcohol dehydrogenase encoding genes, one caffeic acid 3-O-methyltransferase encoding gene, two trans-cinnamate 4-monooxygenase (CYP73A) encoding genes, six phenylalanine ammonia-lyase (PAL) encoding genes, four peroxidase encoding genes, and one gene encoding coniferyl-aldehyde dehydrogenase. PAL, CYP73A and 4CL are all key enzymes in the phenylpropane metabolic pathway. PAL (c3369/f1p14/2256, c11315/f1p23/2741, c1076/f3p52/2334, c14606/f1p46/3127, c87606/f1p10/1076, c68310/f1p8/2095) can catalyze phenylalanine to produce cinnamic acid. Then, through the catalysis of CYP73A (c88562/f1p10/1325, c70286/f1p12/1308) and 4CL (c61149/f1p35/2029, c261046/f2p6/2013, c9430/f1p36/1338), *p*-coumaric acid and *p*-coumaric acid coenzyme A and secondary metabolites are generated (44). N-substituted 3,4-dimethylpyrroles play an important role in the formation of garlic green pigment. We speculated that metabolites in phenylpropane pathway such as phenols will affect the side chain of 2-(1H-pyrrolyl) carboxylic acids, side chains substituted at pyrrole, and conjugated double bonds with two pyrrole rings. Thus affecting the garlic green discoloration. Cold storage stress caused the change of β-glucosidase level (c86756/f1p3/1001, c24450/f1p15/3426, c26268/f1p14/1850, c32513/f1p279/2128), regulated sugar metabolism, and altered the composition of plant cell wall (16). Peroxidase (c44043/f1p3/1144, c12491/f1p3/1162, c22433/f1p2/1336, c317700/f1p2/1273) regulated the contents of ${\text{O}}_{2}^{-}$ and H_2_O_2_ in cells, avoided the membrane damage of plant cells under pressure, and provided conditions for the stability of the structure and function of various organelles in cells (35). These stress-response enzymes encoding genes might be responsible for the discoloration of garlic.

Through KEGG enrichment analysis and GO term enrichment analysis, DEGs of PZ and LW garlic were mainly enriched into phenylpropane metabolic pathway. In addition, DEGs were also enriched in starch and sucrose metabolic pathways, glycolysis pathway, and amino acid biosynthesis pathway. In response to low temperature stress, PZ garlic and LW garlic showed different green variation, and the DEGs of PZ garlic and LW garlic were enriched in phenylpropane metabolic pathway. Phenylpropane metabolic pathway was a defensive response to low temperature, so low temperature may cause garlic green change through phenylpropane metabolic pathway. Starch and sucrose metabolic pathways can affect the amino acid metabolic pathway, which may accelerate the transformation from carbohydrate metabolism to amino acid anabolism and provide precursors for garlic greening (45). Glycolysis oxidizes sugars in organisms to produce ATP, NADH, and pyruvate. The production of ATP makes garlic green. Pyruvate can provide carbon source for the production of amino acids, and then promote garlic greenness stored at low temperature (46).

In addition, the stability of garlic green pigment is primarily affected by light, temperature, pH, and oxygen. Garlic discoloration can be reduced by heat treatment, high-pressure steam treatment, pH adjustment, the addition of chemical additives (47), and freeze-dried fresh onion powder (48).

## Conclusion

In this study, PZ garlic and LW garlic were used as materials to compare the changes of their transcriptional levels during the greening process. This study clarified the mechanism related to garlic greenness and concluded that low temperature may cause garlic greening through phenylpropane metabolic pathway by analyzing the genomes, DEGs, gene function annotation, and metabolic pathways of PZ garlic and LW garlic. Proteomic analysis verified that the results of transcriptomics were consistent with the changes of protein level. In the future, we will detect changes in the content of corresponding metabolites through metabolic analysis. These results would expand our understanding of the mechanism of garlic greenness, and lay a foundation for the following screening and verification of important functional genes, along with revealing the regulation mechanism of gene expression. Moreover, we can inhibit garlic greenness by silencing or over expressing genes in the future.

## Data Availability Statement

The datasets presented in this study can be found in online repositories. The names of the repository/repositories and accession number(s) can be found in the article/Supplementary Material.

## Author Contributions

JXW, ZLN, XML, XZT, and XGQ contributed to the study design, data collection, and data analysis, wrote the first draft, and revised the manuscript. LCM, CL, and NYL were the supervisor of the project and contributed to the study design, data analysis, and manuscript revision. All authors reviewed and accepted the content of the final manuscript.

## Funding

This work was supported by the National Natural Science Foundation of China (31972000) and Major Scientific and Technological Innovation Projects of Key R&D Program of Shandong Province (2019JZZY020607).

## Conflict of Interest

The authors declare that the research was conducted in the absence of any commercial or financial relationships that could be construed as a potential conflict of interest.

## Publisher's Note

All claims expressed in this article are solely those of the authors and do not necessarily represent those of their affiliated organizations, or those of the publisher, the editors and the reviewers. Any product that may be evaluated in this article, or claim that may be made by its manufacturer, is not guaranteed or endorsed by the publisher.

## Supplementary Material

Supplementary Table 1 qRT-PCR Primers of selected unigenes.

Supplementary Table 2 The function of unigenes annotated.

Supplementary Table 3 The all-sample gene expression was standardized by fragments per kilobase of exon per million mapped fragments (FPKM).

Supplementary Table 4 Differentially Expressed Genes (DEGs) identified in samples.

Supplementary Table 5 KEGG pathways enriched by DEGs.

Supplementary Table 6 GO terms enriched by DEGs.

Supplementary Table 7 Differentially Expressed Proteins (DAPs) identified in samples.
